# A randomized, open-label trial of combined nitazoxanide and atazanavir/ritonavir for mild to moderate COVID-19

**DOI:** 10.3389/fmed.2022.956123

**Published:** 2022-09-08

**Authors:** Adeola Fowotade, Folasade Bamidele, Boluwatife Egbetola, Adeniyi F. Fagbamigbe, Babatunde A. Adeagbo, Bolanle O. Adefuye, Ajibola Olagunoye, Temitope O. Ojo, Akindele O. Adebiyi, Omobolanle I. Olagunju, Olabode T. Ladipo, Abdulafeez Akinloye, Adedeji Onayade, Oluseye O. Bolaji, Steve Rannard, Christian Happi, Andrew Owen, Adeniyi Olagunju

**Affiliations:** ^1^Department of Medical Microbiology and Parasitology, University of Ibadan, Ibadan, Nigeria; ^2^Olabisi Onabanjo University Teaching Hospital, Sagamu, Nigeria; ^3^Department of Epidemiology and Medical Statistics, University of Ibadan, Ibadan, Nigeria; ^4^Department of Pharmaceutical Chemistry, Obafemi Awolowo University, Ile-Ife, Nigeria; ^5^State Specialist Hospital, Osogbo, Nigeria; ^6^Department of Community Health, Obafemi Awolowo University Teaching Hospital, Ile-Ife, Nigeria; ^7^Department of Community Medicine, University of Ibadan, Ibadan, Nigeria; ^8^Department of Surveillance and Epidemiology, Nigeria Centre for Disease Control, Abuja, Nigeria; ^9^Oyo State Ministry of Health, Ibadan, Nigeria; ^10^Department of Chemistry, University of Liverpool, Liverpool, United Kingdom; ^11^African Centre of Excellence for Genomics of Infectious Diseases, Redeemer’s University, Ede, Nigeria; ^12^Department of Pharmacology and Therapeutics, University of Liverpool, Liverpool, United Kingdom

**Keywords:** COVID-19, SARS-CoV-2, nitazoxanide (NTZ), atazanavir/ritonavir, pharmacokinetics

## Abstract

**Background:**

The nitazoxanide plus atazanavir/ritonavir for COVID-19 (NACOVID) trial investigated the efficacy and safety of repurposed nitazoxanide combined with atazanavir/ritonavir for COVID-19.

**Methods:**

This is a pilot, randomized, open-label multicenter trial conducted in Nigeria. Mild to moderate COVID-19 patients were randomly assigned to receive standard of care (SoC) or SoC plus a 14-day course of nitazoxanide (1,000 mg b.i.d.) and atazanavir/ritonavir (300/100 mg od) and followed through day 28. Study endpoints included time to clinical improvement, SARS-CoV-2 viral load change, and time to complete symptom resolution. Safety and pharmacokinetics were also evaluated (ClinicalTrials.gov ID: NCT04459286).

**Results:**

There was no difference in time to clinical improvement between the SoC (*n* = 26) and SoC plus intervention arms (*n* = 31; Cox proportional hazards regression analysis adjusted hazard ratio, aHR = 0.898, 95% CI: 0.492–1.638, *p* = 0.725). No difference was observed in the pattern of saliva SARS-CoV-2 viral load changes from days 2–28 in the 35% of patients with detectable virus at baseline (20/57) (aHR = 0.948, 95% CI: 0.341–2.636, *p* = 0.919). There was no significant difference in time to complete symptom resolution (aHR = 0.535, 95% CI: 0.251–1.140, *p* = 0.105). Atazanavir/ritonavir increased tizoxanide plasma exposure by 68% and median trough plasma concentration was 1,546 ng/ml (95% CI: 797–2,557), above its putative EC_90_ in 54% of patients. Tizoxanide was undetectable in saliva.

**Conclusion:**

Nitazoxanide co-administered with atazanavir/ritonavir was safe but not better than standard of care in treating COVID-19. These findings should be interpreted in the context of incomplete enrollment (64%) and the limited number of patients with detectable SARS-CoV-2 in saliva at baseline in this trial.

**Clinical trial registration:**

[https://clinicaltrials.gov/ct2/show/NCT04459286], identifier [NCT04459286].

## Introduction

With over 574 million cases and more than 6.3 million deaths at the end of July 2022 (1), just over 24 months since the first case was reported in mainland China (2), the coronavirus disease 2019 (COVID-19) is so far the second most devastating pandemic since the 1918 flu pandemic (3). More than 2,900 vaccine or therapeutic clinical trials have been registered and hundreds are either completed or ongoing (1, 4, 5).

In a report of *in vitro* studies on the anti-coronavirus activity of 727 compounds in the National Institutes of Health Clinical Collection small molecule library on mouse astrocytoma DBT cells infected with SARS-CoV for 1 h (6), nitazoxanide was among the top three inhibitors, resulting in a reduction of 6 log_10_ in virus titer with an IC_50_ of 1.0 μM. The major circulating metabolite of nitazoxanide is tizoxanide and recent work by the NIH National Centre for Advancing Translational Sciences confirmed its *in vitro* activity against SARS-CoV-2 in Vero E6 host cells *via* suppression of viral cytopathic effect (7). A recent study demonstrated the anti-SARS-CoV-2 activity of nitazoxanide and tizoxanide in a reconstructed bronchial human airway epithelium model (8). We previously identified nitazoxanide among the only 14 drugs able to achieve plasma and lung concentration above the EC_90_ for SARS-CoV-2 at approved doses out of 56 drugs with reported *in vitro* activity (9). In a follow-up study, we explored optimal nitazoxanide dosing schedules for maintaining effective tizoxanide plasma and lung concentrations (10). The susceptibility of 210 seasonal influenza viruses to nitazoxanide and its metabolite tizoxanide has been reported (11) and nitazoxanide reduced symptom duration in acute uncomplicated influenza (12). SARS-CoV-2 shares almost 80% of the genome with SARS-CoV (13) and almost all encoded proteins of SARS-CoV-2 are homologous to SARS-CoV proteins (14). Hence, nitazoxanide and its metabolite tizoxanide with demonstrated *in vitro* activity against SARS-CoV are considered potential candidates for COVID-19.

The HIV protease inhibitor, atazanavir (boosted with ritonavir), has been shown to inhibit the major protease enzyme required for viral polyprotein processing during coronavirus replication (15, 16). It also blocks pro-inflammatory cytokine production (15). Additionally, tizoxanide is inactivated by glucuronidation and atazanavir is a well-known inhibitor (17). Hence, atazanavir is expected to enhance tizoxanide exposure when used in combination with nitazoxanide. Importantly, widespread deployment of antiviral monotherapies for pulmonary viruses (e.g., influenza virus) often leads to the emergence of resistance and we previously called for caution in this regard (18). Therefore, to take advantage of the anticipated favorable drug-drug interaction, a combination of nitazoxanide and atazanavir/ritonavir was selected for this trial.

## Methods

### Study design

The nitazoxanide plus atazanavir/ritonavir for COVID-19 (NACOVID) trial is a pilot open-label randomized phase 2, multicenter, two-arm controlled trial conducted in Nigeria. Patients who recently tested positive for SARS-CoV-2 by means of reverse transcription-polymerase chain reaction (RT-PCR) assay and were symptomatic were eligible. Patients were considered to have a mild disease if they were ambulatory, need little or no assistance. Those with moderate disease were non-ambulatory but had no need for oxygen therapy, or required oxygen by mask or nasal prongs. Severely ill patients that required mechanical ventilation at screening, or had sepsis with end-organ involvement were not eligible. The national guideline for COVID-19 at the time required that all symptomatic cases be managed in isolation and treatment centers established within tertiary hospitals or purpose-built facilities. Hence, the NACOVID trial was conducted in an inpatient setting with participants enrolled after diagnosis and within 48 h of admission.

The National Health Research Ethics Committee, Nigeria (approval number: NHREC/01/01/2007-26/08/2020) and the Central University Research Ethics Committee, University of Liverpool (reference number: 8074) approved the protocol. All patients provided written informed consent as per the ethics committee’s approved process. Further details about the trial design, inclusion and exclusion criteria are provided in the published protocol (19). The trial is registered on ClinicalTrials.gov (NCT04459286) and Pan African Clinical Trials Registry (PACTR202008855701534).

### Randomization

Patients were randomly assigned in a 1:1 ratio to receive either standard of care alone or standard of care combined with 1,000 mg nitazoxanide tablets twice daily and 300/100 mg atazanavir/ritonavir tablets once daily with standard local meal. The selection of these doses was based on three considerations as elaborated for each drug in the published trial protocol (19): (1) demonstration of *in vitro* anti-SARS-CoV-2 activity at doses shown or predicted to be tolerated by humans, (2) the likelihood of achieving effective concentration in relevant compartments, and (3) established human safety record. Randomization was implemented using a Research Electronic Data Capture (REDCap) (20) module that centrally stratified patients by study site, diagnosis CT value, gender, existence of comorbidities and disease severity at enrollment. Standard of care was according to the national interim guidelines for clinical management of COVID-19, including antipyretics for fever, cough medicine, antimalaria in cases with malaria co-infection, multivitamins and mineral supplement, and ongoing treatment of pre-existing comorbidities.

### Procedures

On study day 0 (baseline), patients provided informed consent and were assessed for eligibility. Those who met the eligibility criteria were enrolled and randomized to either continue the standard of care alone (started before study entry in all participants) or trial intervention in addition. The intervention consisted of 1,000 mg nitazoxanide twice daily and 300/100 mg atazanavir/ritonavir administered orally once daily in the night, both administered orally after a meal and directly observed by study staff on days 1–14.

Daily assessment of vitals including SpO_2_, symptom monitoring using the Flu-PRO questionnaire and clinical improvement as well as adverse event monitoring was conducted by designated staff at each study site for all patients on days 1–14, and on days 21 and 28. Saliva for SARS-CoV-2 viral load was collected on days 0, 2, 4, 6, 7, 14, and 28. Saliva and dried blood spots for quantification of tizoxanide, the active metabolite of nitazoxanide, were collected on days 2, 4, 6, 7, and 14 about the same time as viral load samples. All samples were stored on-site at −20^°^C, or lower, and shipped to the testing laboratories: SARS-CoV-2 viral load at the African Centre of Excellence in Genomics of Infectious Diseases (ACEGID), Redeemers University, Ede and pharmacokinetic analysis at the Bioanalytical Laboratory, Obafemi Awolowo University, Ile-Ife, Nigeria. Study data were collected and managed with a 26-form electronic case report form using REDCap (20), a secure, web-based software platform designed to support data capture for research studies hosted at Obafemi Awolowo University.

### Outcomes

The main outcomes were time to SARS-CoV-2 RT-PCR negativity, time to clinical improvement, and temporal patterns of saliva SARS-CoV-2 viral load quantified by RT-PCR. Clinical improvement was defined as the time from randomization to either an improvement of two points on a 10-category ordinal scale or discharge from the hospital, whichever came first (21). Secondary outcomes included time to symptom resolution, clinical status on days 7 and 14 based on the 10-category ordinal scale, day 28 mortality, time from treatment initiation to death and proportion of participants with viral RNA detection over time.

For the assessment of pharmacokinetic endpoints, paired dried blood spots on Whatman 903 protein saver cards (VWR International Ltd., Leicestershire, United Kingdom) and saliva samples were collected to determine the trough concentration of tizoxanide, the active metabolite of nitazoxanide (around 12 h after dose). These were collected on days 2, 4, 6, 7, and 14 at the same time as saliva samples for SARS-CoV-2 viral load. The drug-drug interaction potential of nitazoxanide and atazanavir/ritonavir was investigated in a separate healthy volunteer, two-period cross-over study approved by the Health Research Ethics Committee, Institute of Public Health, Obafemi Awolowo University (IPH/OAU/12/1574). In brief, drug-free healthy volunteers (18–35 years old, male and female) were recruited. Each volunteer received 1,000 mg nitazoxanide 12 hourly after a standard meal for 5 days in the first period, followed by a 21-day washout period. In the second stage, they received 1,000 mg nitazoxanide 12 hourly combined with 300/100 mg atazanavir/ritonavir once daily for 5 days. Plasma samples were collected at 0.25, 0.5, 1, 2, 4, 6, and 12 h after dose on days 1 and 5 during both stages. Tizoxanide quantification was by validated LC-MS/MS methods on TSQ Vantage (Thermo Electron Corporation, Hemel Hempstead, Hertfordshire, United Kingdom) with 50 ng/ml lowest limit of quantification. Data from the first seven participants who completed day 1 of both periods are included in this paper to show the outcome of single-dose interaction. The full study, including an embedded clinical cross validation of the plasma and dried blood spot bioanalytical methods, will be published separately.

### Statistical analysis

A sample size of 98 was estimated to provide more than 80% power to show or exclude 60% improvement in time to SARS-CoV-2 RT-PCR negativity in the intervention group compared with the control group at a two-sided type 1 error rate of 5%. Between-group (SOC vs. Intervention) comparisons of demographic, anthropometric, clinical and laboratory data of the participants were conducted using independent sample *t*-test and Chi-square test of association for continuous and categorical variables, respectively. Analysis of clinical improvement based on the 10-category ordinal scale was performed using the analysis of time-to-event data. Multivariable Cox proportional hazard regression analysis was conducted to assess the differentials in time to improvement. Analysis of cumulative (probability of survival) improvement rate was carried out using Kaplan-Meier survival curves. Primary and secondary outcomes analyses were adjusted for the baseline value of the outcome and randomization stratification factors. SARS-CoV-2 viral load was calculated from the RT-PCR cycle-threshold value. Daily symptom data were aggregated per category (nose and throat, eyes, chest and respiratory, gastrointestinal, and body and systemic) and complete resolution was defined as the disappearance of all abnormalities. Covariates with *p*-value < 0.25 in the univariable Cox regression analysis were included in the multivariable model. These analyses were conducted using Stata Version 17.

## Results

**FIGURE 1:**
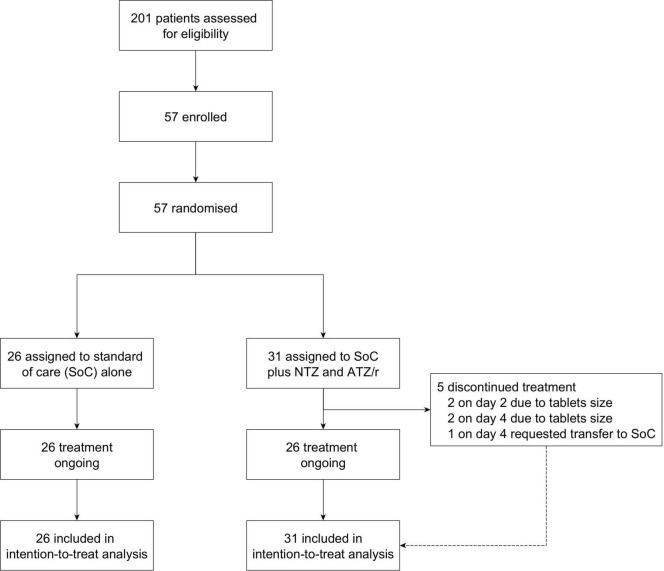
NACOVID trial profile.

The first patient was enrolled on November 25, 2020 and the last patient was enrolled on April 20, 2021. To take advantage of the increasing cases during the second wave of the pandemic in Nigeria, two under-recruiting sites (Obafemi Awolowo University Teaching Hospitals Complex, Ile-Ife and State Specialist Hospital, Osogbo) were withdrawn on February 1, 2021 and a new site (ThisDay Dome COVID-19 Isolation and Treatment Centre, Abuja) was added. However, no patient was enrolled from the new site as the second wave entered the decline phase before ethics and regulatory approvals of the amendments were secured. Hence, only 57 patients were successfully enrolled and randomized from the Infectious Diseases Hospital, Olodo, Ibadan (*n* = 45) and Olabisi Onabanjo University Teaching Hospital, Sagamu (*n* = 12). A total of 26 patients were randomized to the standard of care alone arm and 31 were randomized to the standard of care plus intervention arm (Figure 1). Withdrawn participants data were censored as of the withdrawal date, while those who switched arms were censored as of the day of switching.

### Baseline characteristics

The mean age of patients in the standard of care alone arm was 40 years (standard deviation: 18) and 37 years (13) in the standard of care plus intervention arm. Most participants were male with mean body weights of 67 and 70 kg, respectively. In both arms, about 50% of patients were enrolled within 1–4 days of receiving their diagnosis. All baseline characteristics were similar between both groups (Table 1).

**TABLE 1:** Baseline characteristics of NACOVID trial participants at enrollment.

Participants	All (*N* = 57)	SoC alone (*N* = 26)	SoC with NTZ/ATZ/r (*N* = 31)	*P*-value
Body weight (kg)	68 (11)	67 (11)	70 (11)	0.322
**Body mass index (kg/m^2^)**				
Underweight (<18.5)	3 (5)	2 (8)	1 (3)	0.397
Normal weight (18.5–24.9)	21 (37)	11 (42)	10 (32)	
Overweight (24.5–29.9)	22 (39)	7 (27)	15 (48)	
Obese (≥ 30)	11 (19)	6 (23)	5 (16)	
**Age in years**	38 (16)	40 (18)	37 (13)	0.620
**Number per age group (%)**				
18–50	37 (65)	18 (69)	19 (61)	0.532
51–75	20 (35)	8 (31)	12 (39)	
**Sex**				
Female	19 (33)	7 (27)	12 (39)	0.347
Male	38 (67)	19 (73)	19 (61)	
**Ethnicity**				
Hausa	2 (4)	1 (4)	1 (3)	0.921
Igbo	3 (5)	1 (4)	2 (7)	
Yoruba	44 (77)	21 (81)	23 (74)	
Others	8 (14)	3 (12)	5 (16)	
**Comorbidities**				
No	42 (74)	16 (62)	26 (84)	0.057
Yes	15 (26)	10 (39)	5 (16)	
**Time from diagnosis to enrollment (days)**				
≤1 days	10 (18)	13 (50)	15 (48)	0.468
2–4 days	29 (51)	7 (27)	5 (16)	
≥5 days	18 (31)	6 (23)	11 (36)	
**Disease severity**				
Mild COVID-19	44 (77)	19 (73)	25 (81)	0.571
Moderate COVID-19	10 (18)	6 (23)	4 (13)	
Severe COVID-19	3 (5)	1 (4)	2 (6)	
**Baseline symptoms**				
Nose and throat	57 (100)	26 (100)	31 (100)	1.000
Chest/respiratory	21 (37)	10 (39)	11 (35)	0.816
Gastrointestinal	3 (5)	1 (4)	2 (7)	0.661
Body/systemic	15 (26)	7 (27)	8 (26)	0.924
Ct value at diagnosis	28.4 (6.9)	29.7 (11.2)	29.3 (6.9)	0.338
Saliva SARS-CoV-2 viral load (copies/ml)	127,094 (337,070)	112,256 (325,927)	149,352 (374,849)	0.546
SPO_2%_	97.9 (4)	97.5 (0.64)	98.3 (0.37)	0.263

Data presented as mean (standard deviation, SD) or number (%) and p-values are based on t-test for continuous variables and Chi-square test for categorical variables. Ct, cycle threshold on the reverse transcriptase polymerase chain reaction assay; SARS-CoV-2, severe acute respiratory syndrome coronavirus 2; SPO_2_, peripheral oxygen saturation.

### Primary outcomes

**FIGURE 2:**
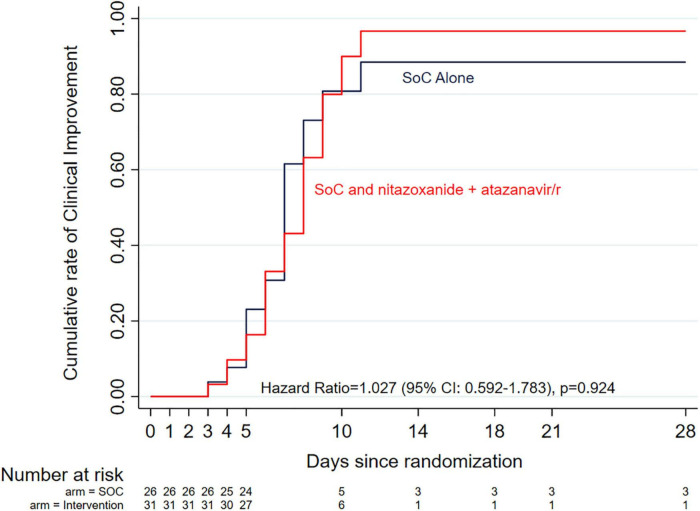
Kaplan-Meier curves of time to clinical improvement (defined as a drop of 2 levels on the 1–10 ordinal scale) by study arm. There was no difference between the two arms (7 days in the standard of care arm alone vs. 8 days in the standard of care plus intervention arm). The Cox proportional hazards model adjusted hazard ratio was 0.898 (95% Cl: 0.492–1.638, *p* = 0.725) after adjusting for potential co-founders, including randomization stratification variables, age and sex.

At the time of enrollment, 19 of the 26 patients randomized to the standard of care alone arm had mild disease (grades 1–3) and 6 had moderate disease (grades 4–5). Of the 31 patients randomized to the standard of care plus intervention, disease severity was mild in 25 and moderate in 4 patients. Three patients who required high flow oxygen (grade 6 severe disease) were enrolled (1 in standard of care alone arm, 2 in standard of care plus intervention arm). The time to achieve protocol-defined clinical improvement (a drop of 2 levels on the 1–10 ordinal scale) in the entire cohort was 7 days and no difference was observed between the two arms (7 days in the standard of care arm alone vs. 8 days in the standard of care plus intervention arm). The hazard ratio (HR) was 1.027 (95% CI: 0.592–1.783), *p* = 0.924 and no difference was observed after adjusting for potential co-founders in Cox proportional hazards regression analysis, including randomization stratification variables (aHR = 0.898, 95% CI: 0.492–1.638, *p* = 0.725; Figure 2). In a separate analysis, we further explored time to clinical improvement in various subgroups using logrank tests but found no significant differences between both arms (Supplementary Table 1).

**FIGURE 3:**
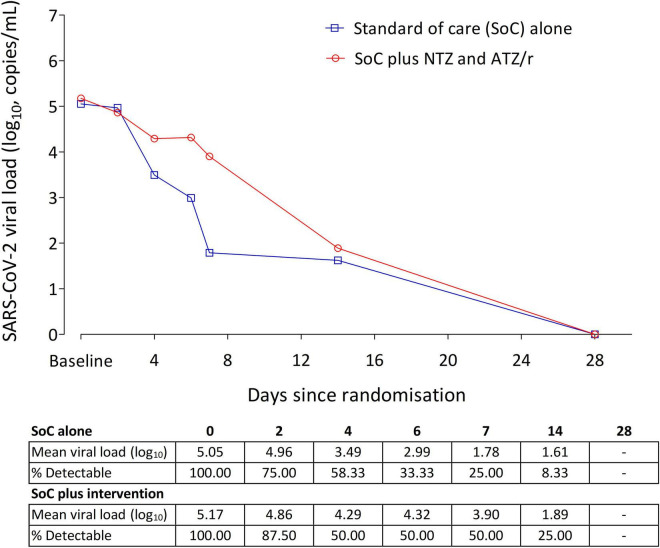
Changes in SARS-CoV-2 viral load in saliva of patients from enrollment to study day 28. In the 20 patients with detectable saliva viral load at enrollment, baseline viral load was 5.05 log10 copies/ml in the SoC alone arm (*n* = 12), and 5.17 log10 copies/ml in the SoC plus intervention arm (*n* = 8). In this small cohort, there was no difference in the rate of viral load decline between the two arms (Cox proportional hazards model aHR = 0.948, 95% Cl: 0.341–2.636, *p* = 0.919).

SARS-CoV-2 was detectable in saliva samples collected at enrollment only in 35% (20/57) of patients with a mean of 5.05 log_10_ copies/ml in SoC alone arm, and 5.17 log_10_ copies/ml in SoC plus intervention arm. In a very limited analysis of this outcome using days 2, 4, 6, 7, 14, and 28 follow up saliva viral load data from these patients, there was no trend toward a difference in the pattern of viral load changes between the two arms, Welch’s *t*-test *p*-value = 0.758 for comparison of means over the follow-up period (Figure 3). The aHR was 0.948 (0.341–2.636) with a *p*-value of 0.919.

### Secondary and safety outcomes

**FIGURE 4:**
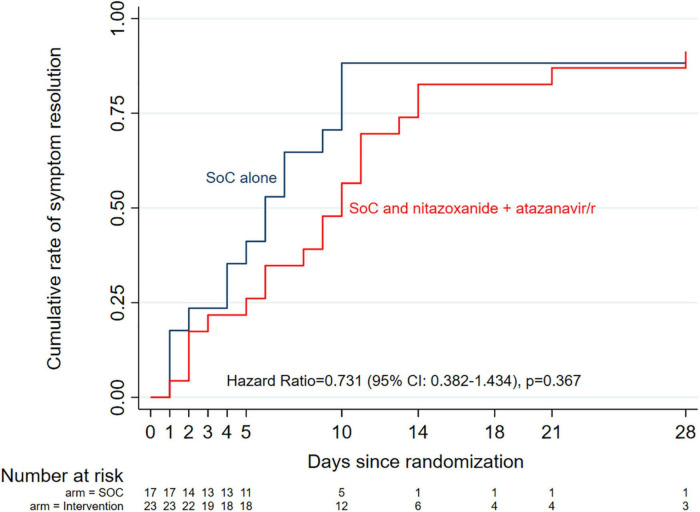
Kaplan-Meier curves of median time to complete symptom resolution by study arm. Overall, there was no significant difference between the two arms, even after adjusting for potential co- founders (Cox proportional hazards model aHR = 0.535, 95% Cl: 0.251–1.140, *p* = 0.105).

The median (range) time from enrollment to complete symptom resolution was 8 (6–14) days in the entire cohort, with a non-significant trend (Kaplan Meier HR = 0.617 (95% CI: 0.311–1.224, *p* = 0.167) toward a shorter time in the standard of care alone arm (6 days) compared with standard of care plus intervention arm (10 days) (Figure 4). Multivariable Cox proportional hazards regression analysis adjusting for randomization variables showed a similar overall non-significant trend (aHR = 0.535, 95% CI: 0.251–1.140, *p* = 0.105), except for disease severity where moderately ill patients were 67% more likely to achieve complete symptom resolution if they received standard of care alone compared with standard of care plus intervention (aHR = 0.322 (95% CI: 0.122–0.848, *p* = 0.022). Further exploration of median time to complete symptom resolution in various subgroups using logrank tests showed no trend toward any benefit in combining the intervention with the standard of care (Supplementary Table 2).

The DSMB at their meeting of 14 November 2021 recommended terminating the trial as no further opportunities existed to recruit additional patients and accrued data did not indicate any trend of benefit in adding the intervention to the standard of care.

Nitazoxanide (1,000 mg b.i.d.) combined with the usual dose of atazanavir/ritonavir (300/100 mg od) was well tolerated in this cohort. Laboratory values of hematology and blood chemistry parameters on days 0, 7, and 14 were within normal ranges (Supplementary Table 3) with no deviations qualifying as grade 1–4 adverse events. In the standard of care plus intervention arm, six patients reported transient known side effects of study drugs (urine discoloration in four and mild abdominal pain in two). No other clinical adverse event was reported.

### Pharmacokinetics of nitazoxanide active metabolite in coronavirus disease 2019 patients

**FIGURE 5:**
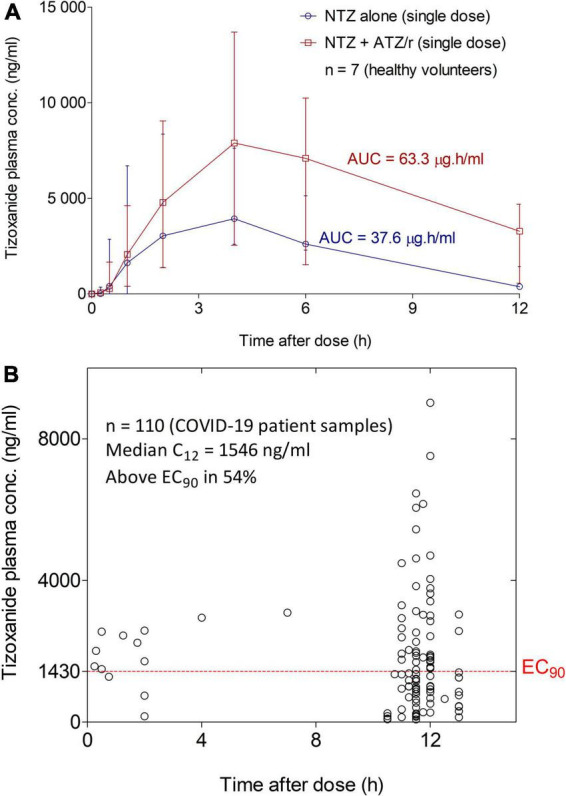
Tizoxanide Concentration-time profiles in healthy volunteers and plasma concentration in COVID-19 patients. **(A)** Co-administration of nitazoxanide (NTZ) with atazanavir/ritonavir (ATZ/r) increased plasma tizoxanide AUC0-12 by 68.3% (37.6μg.h/ml vs. 63.3 μg.h/ml) and its Cmax by 14.4% (7,630 ng/ml vs. 8,730 ng/ml). **(B)** Using samples collected at 11–12 h after the last nitazoxanide dose (1,000 mg b.i.d.), the median concentration was 1,546 ng/ml, above the EC90 of SARS-COV-2 in 54% of patient samples.

We compared concentration-time data from day 1 of both periods of the drug-drug interaction study from seven healthy volunteers: 4 females and 3 males aged 24.4 years (4.8) with 56.6 kg (7.5) body weight. Co-administration of nitazoxanide (NTZ) with atazanavir/ritonavir (ATZ/r) increased plasma tizoxanide median (range) AUC_0–12_ by 68.3% from 37.6 μg.h/ml (19.9–45.9) to 63.3 μg.h/ml (54.3–84.6). Also, the median (range) C_*max*_ by 14.4% from 7,630 ng/ml (2,600–9,490) to 8,730 ng/ml (7,230–14,271) (Figure 5A). A total of 110 concentration-time data were available from the 31 patients in the standard of care plus intervention arm. Median (range) tizoxanide trough (around 12 h after nitazoxanide dose) plasma concentration was 1,546 ng/ml (95% CI: 797–2557), above its putative EC_90_ in 54% of patients (22) (Figure 5B). An EC_90_ of 1,430 ng/ml was reported for nitazoxanide in reversing SARS-CoV-2 induced cytopathic effect in Vero E6 host cells, and tizoxanide is expected to have a similar *in vitro* potency (7). Tizoxanide was undetectable in saliva samples collected in the drug-drug interaction study and from patients.

## Discussion

In this pilot randomized open-label trial, patients who received a 14-day course of nitazoxanide (1,000 mg b.i.d.) and atazanavir/ritonavir (300/100 mg od) in addition to standard of care initiated within a few days of COVID-19 diagnosis did not experience a better outcome (clinical improvement, viral clearance, and symptom resolution) compared with those who received standard of care alone. Crucially, tizoxanide plasma exposure was significantly enhanced when combined with atazanavir/ritonavir as expected, possibly *via* inhibition of its inactivation through glucuronidation (17). Though concentration in patients at 12 h after dose was lower than in healthy volunteers, an observation that may be due to the influence of certain components of standard of care, it was above the putative tizoxanide plasma EC_90_ in more than 50% of patients. This is similar to the achievement of plasma concentration above the EC_90_ in 51% of virtual subjects given 1,000 mg b.i.d. nitazoxanide with food (10). However, tizoxanide was undetectable in saliva samples collected from participants in the drug-drug interaction study and in patients throughout the follow-up period. Tizoxanide is highly bound to plasma proteins (over 99.9%) and we previously highlighted the critical importance of this parameter for *in vitro* to *in vivo* extrapolation (23). Our predictions of tizoxanide distribution to human lung (9, 10) based on physicochemical properties, *in vitro* drug binding information, and tissue-specific data did not accurately recapitulate *in vivo* observation.

Several ongoing, completed, or terminated clinical trials include nitazoxanide as monotherapy or as part of a combination strategy. A preprint of interim analysis from a study by Silva et al. (clinicaltrials.gov identifier: NCT04463264; *n* = 45) showed no difference in the achievement of PCR negativity by day 7 (62.5% of patients in the 500 mg q.i.d. nitazoxanide arm vs. 53.9% in the placebo arm, *p* = 0.620) (24). In the Vanguard study (NCT04486313, *n* = 379) that enrolled outpatients with mild or moderate COVID-19 within 72 h of symptom onset, 600 mg b.i.d. extended release nitazoxanide was reported to reduce progression to severe COVID-19 by 85% (1/184, 0.5%) compared with placebo (7/195, 3.6%; *p* = 0.07). There was no overall difference in time to sustained clinical recovery (25). Elalfy et al. study (NCT04392427, *n* = 113) reported a cumulative day-15 SARS-CoV-2 clearance rate of 88.7% in patients with mild COVID-19 who were treated with a combination of nitazoxanide (500 mg q.i.d.), ribavirin, and ivermectin plus zinc supplement compared with 13.7% in those who received supportive symptomatic therapy (no data on statistical significance) (26). Understandably, it is uncertain which particular agent in the combination is responsible for the observed efficacy in the latter study. In the SARITA-2 study (NCT04552483, *n* = 392), PCR negativity was achieved in 29.9% of patients who received nitazoxanide (500 mg t.i.d. for 5 days) compared with 18.2% in the placebo arm (*p* = 0.009) and 55% reduction in viral load compared with 45% (*p* = 0.013). However, there was no difference in symptom resolution between the nitazoxanide and the placebo arms (27). Taken together, all three studies where viral load was evaluated reported some benefit, both studies that evaluated symptom resolution observed no benefit, while both studies that evaluated PCR negativity reported conflicting findings.

Similar to the Vanguard and SARITA-2 studies, data from the NACOVID study showed no difference in clinical improvement or symptom resolution between patients treated with standard of care alone vs. standard of care plus nitazoxanide (1,000 b.i.d.) and atazanavir/ritonavir. However, we only achieved 64% of the target sample size of 89 required to show or exclude 60% improvement in time to SARS-CoV-2 PCR negativity (19). Additionally, the limited number of patients with detectable SARS-CoV-2 in saliva at baseline requires that our finding of no difference in viral load change in this trial be interpreted with caution. The choice of saliva for SARS-CoV-2 viral load in this trial was based on observed concordance with nasopharyngeal swabs in the testing laboratory and similar early reports (28, 29). More recent data now suggest that the suitability of saliva as an alternative to nasopharyngeal swab may be limited to disease stages associated with high viral load (30, 31). Unfortunately, delays in pre-enrollment testing and diagnosis may have resulted in most patients entering the trial after the exponential phase. Crucially, COVID-19 diagnosis in Nigeria was limited to symptomatic cases and some asymptomatic individuals (e.g., arrival from a COVID-19 hotspot country) during the trial. Hence, immunity acquired from previous undiagnosed asymptomatic infection is likely to contribute to quicker viral clearance in subsequent infections even in the absence of treatment. Unfortunately, the time to symptom onset was not available in our baseline data. Hence, the stage of disease the patients were in at enrollment (early disease with lots of viral dynamics, or later stage with continuous clearance of virions) was not known and this is a limitation. An additional limitation was the possibility of missing out certain crucial viral measurement on days 1, 3, and 5 within the first 7 days of participation.

The absence of detectable levels of nitazoxanide active metabolite tizoxanide in saliva samples in this may be indicative of poor penetration into this matrix. If confirmed, this underscores some important points. The use of plasma as a surrogate for target site concentration in COVID-19 should be supported by confirmation of adequate penetration into the respiratory tract and acceptable correlation as with certain antituberculosis drugs (32), Remdesivir is known to penetrate poorly into human lungs after intravenous administration (33), and nebulized formulation is currently under development (34) to further enhance its *in vivo* efficacy. Inhalation delivery with targeted activation within the lungs (35, 36) will be an important strategy for drugs with confirmed *in vitro* activity against SARS-CoV-2 but poor penetration into human lungs.

In a computational model of human SARS-CoV-2 viral kinetics with acquired immune response, doses of nitazoxanide as high as 2,900 mg twice daily was found to have no appreciable effect. The study also highlighted the critical importance of early initiation of treatment (37). Reports from other completed clinical studies are pending while several others are still recruiting, including a phase Ib/IIa study investigating within the AGILE clinical trial platform (NCT04746183) (38) the efficacy of the 1,500 mg b.i.d. dosage which was shown to be safe with acceptable tolerability (39) in mild to moderate COVID-19. As it is unlikely that doses higher than 1,500 mg b.i.d. will be tolerable, the AGILE trial is expected to provide a firm signal for whether efficacy can be achieved in COVID-19 at any dose.

## Data availability statement

The original contributions presented in this study are included in the article/Supplementary material, further inquiries can be directed to the corresponding author.

## Ethics statement

The studies involving human participants were reviewed and approved by the National Health Research Ethics Committee, Nigeria (approval number: NHREC/01/01/2007-26/08/2020), the Central University Research Ethics Committee, University of Liverpool (reference number: 8074), and the Health Research Ethics Committee, Institute of Public Health, Obafemi Awolowo University (approval number: IPH/OAU/12/1574). The patients/participants provided written informed consent to participate in this study.

## Author contributions

AdOn, AnO, and SR conceived the initial study. AdOn designed the study and developed the protocol with input from all authors. AF, FB, BE, and BOA were responsible for study enrollment and data acquisition. CH was responsible for SARS-CoV-2 viral load determination using RT-PCR. AdOn, AbA, BA, and OOB were responsible for database management and pharmacokinetic analyses. AdOn, AbA, AF, and BE verified the underlying data. AFF and AdOn were responsible for analysis and interpretation of data. AdOn drafted the manuscript. AnO and OOB critically revised the manuscript. All authors contributed to conducting the trial, revised the report and read and approved the final version before submission, had full access to all the data in the study, and had final responsibility for the decision to submit for publication.
